# The Role of the C3a‐C3aR Pathway in Diseases: Latest Research Advances

**DOI:** 10.1155/mi/1754881

**Published:** 2026-04-17

**Authors:** Xiangwei Bo, Mi Wang, Yang Liu, Yong Zhong

**Affiliations:** ^1^ Department of Health Medicine, Affiliated Jinling Hospital, School of Medicine, Nanjing University, Nanjing, 210002, China, nju.edu.cn; ^2^ Department of Cardiology, Zhongda Hospital, School of Medicine, Southeast University, 87 Dingjiaqiao, Nanjing, 210009, China, seu.edu.cn; ^3^ Department of Critical Care Medicine, Affiliated Jinling Hospital, School of Medicine, Nanjing University, Nanjing, 210002, China, nju.edu.cn

**Keywords:** autoimmune diseases, C3a-C3aR pathway, cancer, cardiovascular diseases, inflammation, kidney diseases, neurodegenerative diseases, therapeutic target

## Abstract

C3a is a key active factor in the complement system, capable of participating in various physiological and pathological processes by binding to its C3a receptor (C3aR). The C3a‐C3aR pathway not only plays an important role in immune regulation but is also closely related to the occurrence and development of various inflammatory diseases, cardiovascular diseases, autoimmune diseases, and tumors. In recent years, research on the specific mechanisms of action of C3a‐C3aR in different diseases has gradually deepened. This article aims to review the basic functions of the C3a‐C3aR pathway, with a focus on summarizing the latest research advances in kidney diseases, cardiovascular diseases, neurodegenerative diseases, autoimmune diseases, and tumors, and to explore its prospects as a potential therapeutic target.

## 1. Introduction

The complement system is an important component of the innate immune defense, with C3a being a small peptide fragment generated by the cleavage of C3. C3a mediates a series of immune and inflammatory responses through its C3a receptor (C3aR), possessing the function of chemotaxis and activation of various immune cells. Under normal circumstances, C3a plays a protective role by promoting host defense against pathogens; however, under certain pathological conditions, excessive activation of the C3a‐C3aR pathway may trigger adverse reactions, leading to tissue injury, chronic inflammation, and exacerbation of diseases. In recent years, with the in‐depth study of complement biology, the pathological role of C3a‐C3aR in various diseases has been gradually revealed, becoming a significant research focus. This review aims to systematically survey the latest research on the C3a‐C3aR pathway across different disease fields, critically synthesize seemingly contradictory findings, and for the first time propose an integrative “context‐dependent determinants” framework to explain its functional variability. We emphasize that any discussion of this pathway, from its basic biology to therapeutic application, must be framed within specific spatiotemporal and pathological microenvironments.

## 2. Basic Biology and Signaling Complexity of the C3a‐C3aR Pathway

### 2.1. Classical Complement Activation and Basic Biology of the C3a‐C3aR Pathway

**Figure 1:**
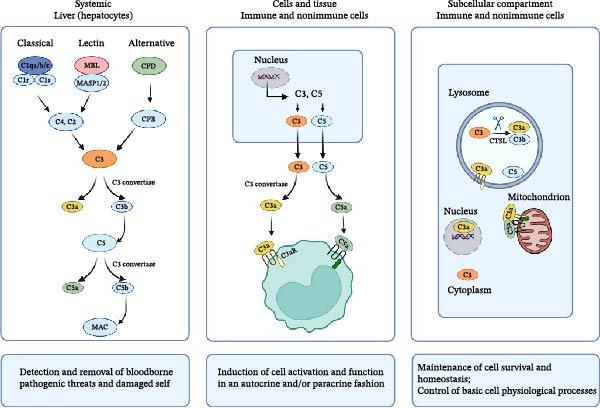
The complement system and its functional compartmentalization. Circulating liver‐produced complement can be activated through three pathways that result in the formation of C3 and C5 convertases, in which cleavage activates C3 into C3a and C3b and C5 into C5a and C5b, respectively. This process leads to the formation of the membrane attack complex (MAC) and the induction of classical complement functions. Activation of cell‐autonomous, intracellular complement in immune and nonimmune cells can occur at different subcellular locations. C3aR, C3a receptor; C5aR, C5a receptor; CTSL, cathepsin L; MAC, membrane attack complex; MASP1/2, MBL serine proteases 1 and 2; MBL, mannose‐binding lectin.

Complement proteins in the circulatory system usually exist in an inactive state and only become active under specific conditions. The three activation pathways are ① the classical pathway, initiated by the binding of C1q to two or more IgG1‐3 or IgM antibodies that have bound to antigens; ② the alternative pathway, which is the most evolutionarily ancient pathway, directly activates C3 by recognizing microbial surface structures and is initiated after forming C3 convertase with the involvement of Factor B and Factor D; ③ the lectin pathway (mannose‐binding lectin [MBL] pathway), triggered by MBL in the plasma directly recognizing carbohydrates on the surfaces of pathogens or the glycosylation of the IgG4 Fc segment, leading to a cascade reaction forming the same C3 and C5 convertases as the classical pathway, activating the complement cascade enzyme‐promoted reaction activation pathway [1, 2]. The three aforementioned pathways converge at the central molecule, C3, in the central step of complement activation [3]. C3a is generated by the cleavage of complement C3 through C3 convertase and primarily functions by binding to the G protein–coupled receptor C3aR [3, 4]. C3aR is widely expressed on various cell types, including monocytes [5], macrophages (MPs) [6], neutrophils [5, 7], endothelial cells [8], podocytes [9], and microglia [10, 11]. After binding with C3aR, C3a regulates inflammation, chemotaxis, and immune responses through downstream signaling pathways such as the PI3K‐Akt and MAPK pathways [11]. C3a‐C3aR not only plays an important role in infection defense but is also closely related to various noninfectious diseases [4] (Figure 1).

### 2.2. Intracellular Complement System

In recent years, scientists have pioneered the discovery of the intracellular complement system in T cells, extending complement research into the intracellular domain, where intracellular C3 can also be cleaved by cathepsin L (CTSL) into C3a and C3b, some of which may be released outside the cell [12]. This high‐order protein complex, distinct from extracellular complement, was defined as the “complosome” in 2016 [13]. Complement subunits are a series of complement molecules inherent to the cell and active within it, with encoding genes consistent with those from the liver and present in the bloodstream. They play important roles in cellular physiological activities that were previously unrecognized. Intracellular complements are found in both immune and nonimmune cells, with current research focusing primarily on the functions of C3 and C5. Complement subunits are mainly located in the cytoplasm, lysosomes, endoplasmic reticulum, outer mitochondrial membrane [14, 15], and nucleus of cells. They can interact with each other and also with intracellular danger sensors and effector systems, but their functions differ from those of complements in the circulatory system [16, 17] (Figure 1). This provides a new dimension for understanding the role of C3aR signaling in cell‐autonomous homeostasis while also explaining its functional basis in noninflammatory states such as neural plasticity and tissue repair.

### 2.3. A Core Concept: Context‐Dependent Determinants of Functional Output

The “double‐edged sword” effect of the C3a‐C3aR signaling pathway in diseases is fundamentally governed by its highly context‐dependent functional output. This property can be systematically explained by an integrative framework, wherein the net effect of the pathway is determined by the collective interplay of four key dimensions: time, cell type, trigger, and disease stage.

The temporal dimension delineates the critical window of action: Signaling in the acute phase often exacerbates inflammatory damage, whereas in the repair phase, it shifts towards promoting tissue remodeling and neural plasticity. The cellular dimension serves as the decoding hardware: C3aR activation on myeloid immune cells primarily mediates host defense and inflammatory regulation, whereas its persistent activation on parenchymal cells tends to drive pathological processes like fibrosis. The trigger dimension defines the nature of the signal: Activation triggered by PAMPs typically directs protective immune responses, while engagement by DAMPs or autoantigens is frequently linked to chronic inflammation and tissue destruction. Finally, the disease stage dimension captures dynamic progression: The pathway can evolve from a participant in disease initiation to a key driver sustaining later‐stage progression.

These four dimensions are interconnected, collectively shaping the ultimate output of the C3a‐C3aR signaling network. Understanding this context‐dependency framework is the theoretical cornerstone for moving beyond a simplistic view of C3a as a static proinflammatory mediator and advancing towards precision interventions with temporal and cellular specificity.

## 3. Role of the C3a‐C3aR Pathway in Diseases

### 3.1. Kidney Diseases: The Paradox From Progressive Fibrosis to Infection Defense

The levels of C3a and C3aR are elevated in various types of kidney diseases and are associated with progression and severity. In the kidney, C3aR is primarily expressed on renal tubular epithelial cells. The C3a/C3aR pathway promotes the progression of glomerular and tubulointerstitial diseases, while it has a protective effect against urinary tract infections.

**Figure 2:**
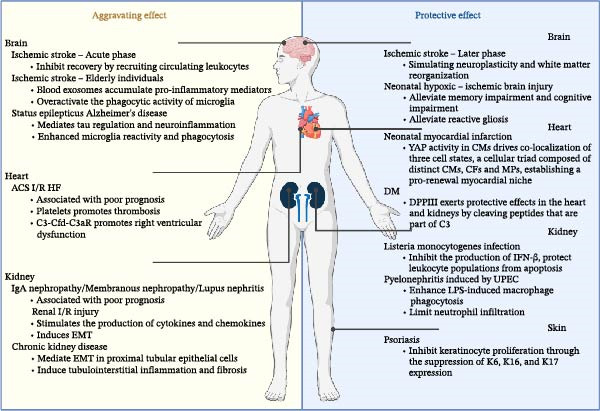
Roles of C3a/C3aR signaling pathway in aggravating effect and protective effect in disease. The C3a‐C3aR signaling pathway has aggravating and protective effects in the brain, heart, kidney, and autoimmune diseases and exhibits the characteristics of double‐edged sword. C3aR, C3a receptor; CM, cardiomyocytes; CFs, cardiac fibroblasts; EMT, epithelial‐mesenchymal transition; I/R, ischemia reperfusion; LPS, lipopolysaccharide; MPs, macrophages; UPEC, uropathogenic *Escherichia coli*.

Clinical studies have shown that in patients with IgA nephropathy, C3aR and C3a staining in the glomeruli are elevated, and C3a levels in serum and urine are also increased [18]. Urinary C3a levels, along with glomerular C3aR and C3a staining, correlate positively with proteinuria, serum creatinine levels, and histopathological injury [19]. In a mouse model of IgA nephropathy induced by Sendai virus, C3aR gene knockout mice showed reduced IgA and C3 deposition in the kidneys, lower levels of proteinuria, decreased proinflammatory cytokines, and reduced histological damage [20]. While the underlying mechanisms were not explored in depth by the authors, this protective effect observed in C3aR/C5a receptor (C5aR) deficiency may be partly attributed to the suppression of cytokine and chemokine expression in the kidneys. In mouse models of lupus nephritis and rat models of diabetic nephropathy, treatment with C3aR antagonists (C3aRAs) has been shown to reduce the expression of inflammatory cytokines in the kidney, alleviate pathological damage, and improve survival. In lupus nephritis, C3aRA treatment led to decreased renal mRNA levels of IL‐1β and RANTES, reduced protein levels of phosphorylated PTEN (phosphatase and tensin homolog deleted on chromosome 10), and increased levels of phosphorylated Akt/protein kinase B, thereby mitigating pathological injury and extending survival [21]. In diabetic nephropathy, C3aRA activated the PI3K/Akt/FoxO1 pathway, which in turn promoted mitochondrial biogenesis and mitophagy in podocytes, helping to maintain podocyte homeostasis and offering a potential targeted strategy against diabetic kidney disease [22]. Patients with membranous nephropathy exhibit markedly elevated levels of C3a in both plasma and urine, with plasma C3a levels decreasing upon disease remission [23]. Furthermore, multiple studies indicate that C3a/C3aR signaling contributes to the pathogenesis of focal segmental glomerulosclerosis (FSGS) by regulating various signaling pathways in glomerular and tubular cells. C3a promotes the transcription of versican by activating the Akt/β‐catenin pathway. The resulting increase in tubule‐derived versican V1 activates fibroblasts, thereby promoting interstitial fibrosis in FSGS [24]. Given these mechanisms, C3aRAs hold therapeutic potential, and targeted therapy against the C3a‐C3aR signaling pathway may be applicable for patients with FSGS [21, 24] (Figure 2).

In renal ischemia‐reperfusion injury, C3a‐C3aR stimulates the production of cytokines and chemokines [25], promotes the expression of the functional and specific marker KIM‐1 of acute tubular necrosis, and participates in kidney injury [26]. In addition, C3a induces epithelial‐mesenchymal transition (EMT) by enhancing NADPH oxidase activity and promoting α‐SMA protein expression [26], thereby regulating IR renal injury and fibrosis [27]. In chronic kidney disease, C3a‐C3aR can mediate EMT in proximal tubular epithelial cells through the TGF‐β1/CTGF signaling pathway, inducing tubulointerstitial inflammation and fibrosis; blocking this pathway can protect renal function and limit interstitial fibrosis [28, 29] (Figure 2).

Contrary to previous findings, the C3a‐C3aR signaling pathway plays a protective role during infection. Studies have demonstrated that C3aR^-/-^ mice infected with *Listeria monocytogenes* exhibit decreased survival, increased bacterial burden, and aggravated liver and spleen injury, which is attributable to a marked increase in apoptosis of both myeloid and lymphoid cells in the spleen. The C3a‐C3aR signaling pathway mediates protection against *L. monocytogenes* infection through a dual mechanism. On one hand, it directly inhibits immune cell apoptosis by downregulating Fas expression, suppressing caspase‐3 activation, and upregulating Bcl‐2 expression, thereby preserving effector cell populations [30]. On the other hand, it modulates intracellular pathogen‐sensing pathways: Binding of C3a to its receptor C3aR, dependent on signaling molecules such as Bruton’s tyrosine kinase, p38 MAPK, and TBK1, suppresses the production of IFN‐β triggered by cyclic di‐AMP (c‐di‐AMP), thereby mitigating immune injury caused by excessive IFN‐β [31]. This protective role is clearly demonstrated in a model of uropathogenic *Escherichia coli* (UPEC)–induced pyelonephritis. Following infection, C3aR^-/-^ mice develop more severe renal injury and higher bacterial burden and are accompanied by excessive production of proinflammatory mediators and neutrophil infiltration, indicating a disruption of inflammatory regulation. The core of the protective mechanism lies in C3aR expression on myeloid cells, such as MPs, which operates through a dual pathway: first, by suppressing the excessive proinflammatory response triggered by ligands like LPS, thereby mitigating immunopathological damage; and second, by directly enhancing the phagocytic capacity of these cells to clear bacteria. Bone marrow chimera experiments confirmed that the absence of C3aR specifically on myeloid cells is key to the exacerbated infection, whereas pharmacological activation of this pathway can effectively improve the pathological outcome [32] (Figure 2).

The critical question is: Why do anti‐infection protection and autoimmune damage coexist within the same kidney? We hypothesize that cell type–specific signal decoding is central to this phenomenon. C3aR signaling in parenchymal cells under chronic stimulation tends to couple more readily with profibrotic pathways (e.g., TGF‐β), whereas C3aR signaling in myeloid cells may be biased toward activating antiapoptotic (e.g., Bcl‐2) and phagocytic programs. Additionally, disease stage (acute infection vs. chronic autoimmunity) and local complement activation levels serve as important modulating factors. Future studies should employ cell‐specific knockout models to validate this hypothesis.

### 3.2. Cardiovascular Disease: Balancing Inflammation, Thrombosis, and Repair

The C3a‐C3aR pathway plays a multifaceted and pivotal role in cardiovascular diseases, spanning multiple pathological processes including atherosclerosis development, acute ischemic events, and the progression of heart failure. Clinical evidence indicates a close association between complement system activation and cardiovascular risk: Serum C3 level is an important independent risk factor for coronary heart disease, and both C4 levels and the C3/C4 ratio hold predictive value for cardiovascular events [33, 34]. In patients with acute coronary syndrome and unstable angina, complement activation levels are significantly elevated, suggesting their involvement in the unstable progression of these conditions.

The role of this pathway is particularly prominent in acute myocardial infarction and reperfusion injury. After reperfusion in humans, the degree of deposition of complement activation products C3 and C5b‐9 in myocardial tissue is significantly increased, while complement depletion caused by complement deficiency or cobra venom factor can significantly reduce the area of myocardial tissue injury, with almost no deposition of C3 in the infarcted area [35]. The C3a‐C3aR pathway plays an important role in cardiovascular diseases, especially in the inflammatory response and tissue repair following myocardial infarction. Hill and Ward [36] found in rat experiments that cardiac tissue can release C3a fragments after MI. C3a, as a bioactive peptide, has a strong chemotactic effect on monocytes [37–39]. Mechanistically, C3a recruits inflammatory cells to the ischemic area through its potent chemotactic effect on monocytes, thereby driving the inflammatory response. Furthermore, in patients with coronary artery disease, platelet complement C3aR expression positively correlates with activated GPIIb/IIIa, and C3aR coexpresses with GPIIb/IIIa in thrombi of myocardial infarction patients. The C3a‐C3aR signaling pathway on platelets promotes thrombus formation and facilitates hemostasis following vascular injury and in vivo thrombosis. Notably, genetic deficiency of C3aR reduces the incidence of myocardial infarction and stroke in mice, revealing its pivotal role in thrombotic events [40] (Figure 2).

This pathway is also involved in the pathophysiology of heart failure and the process of ventricular remodeling. The level of C3a in patients with acute heart failure is higher than in healthy controls; however, in age‐adjusted subgroup analyses, there is no difference in the level of C3a between AHF patients and the control group, and it remains constant during hospitalization [41]. At the same time, C3a is a significant predictor of heart failure‐related rehospitalization or death and cardiovascular events or death [42]. Research by Shogo Ito and colleagues has revealed the critical role of the complement system in right ventricular failure. Knockout of C3 and Cfd genes and the use of C3aRAs can improve right ventricular dysfunction caused by pulmonary artery constriction in mice. The C3‐Cfd‐C3aR signaling axis is a core mechanism of right ventricular failure [43].

Notably, under specific regenerative or metabolic conditions, C3a‐C3aR pathway may also exhibit protective effects. A study from the Texas Heart Institute in the United States shows that under proregenerative conditions, there is a close interaction between specific myocardial cell subtypes (aCM2), fibroblasts expressing complement C3, and MPs expressing C3ar1. For example, there is increased expression of the active form of YAP in myocardial cells of regenerative neonatal hearts and adult mouse hearts [44]. In animal models of DM, DPPIII exerts protective effects in the heart and kidneys by cleaving peptides that are part of C3 and through its interaction with C3aR and protein kinase C–mediated RhoA activation downstream of the receptor in endothelial cells [45] (Figure 2).

In summary, the C3a‐C3aR pathway primarily exerts proatherosclerotic, prothrombotic, ischemia‐reperfusion injury‐aggravating, and adverse ventricular remodeling–promoting effects in cardiovascular diseases. However, its functions may exhibit complexity depending on specific pathological contexts, such as disease stage and the presence of regenerative conditions. This provides a rationale for precision therapy targeting this pathway and suggests that interventional strategies must be highly tailored to the specific disease context.

### 3.3. Neurological Diseases: Temporal Regulation From Acute Neurotoxicity to Long‐Term Plasticity

The role of the C3a‐C3aR pathway in central nervous system diseases profoundly illustrates its extreme temporal dependency and pathological context specificity. This characteristic is most clearly elucidated in ischemic stroke and serves as the basis for understanding its complex manifestations in other neurological disorders.

In ischemic stroke, the temporal dimension is central to the functional determination of this pathway. It is noteworthy that this peripheral‐central detrimental interplay is further amplified in the context of aging: Studies indicate that exosomes in the blood of elderly individuals can carry and accumulate peripheral proinflammatory mediators, cross the blood‐brain barrier via a C3aR‐dependent mechanism, and subsequently hyperactivate the phagocytic activity of microglia, ultimately exacerbating the poor outcomes of ischemic stroke [46]. In contrast, upon entering the subacute phase (approximately after day 7), the same signaling cascade transforms into a driver of neural repair. C3a inhibits the excessive activation of astrocytes, upregulates plasticity factors such as insulin‐like growth factor‐1 (IGF‐1) and thrombospondin 4 (THBS4), and promotes cerebral white matter remodeling and cortical neural connection reconstruction. Meanwhile, C3a intervention at this stage does not introduce harmful inflammatory cells or abnormal microglia, can prevent microglia from transforming into the proinflammatory phenotype, reduce neutrophil infiltration, and avoid inflammation‐mediated secondary brain damage, thereby accelerating neural function recovery.

The core mechanism underlying this transition lies in the intrinsic plasticity of astrocytes and their interaction with the dynamically evolving microenvironment. This reveals a critical therapeutic paradigm: Interventions targeting C3aR (e.g., intranasal administration of C3a between days 7 and 30 poststroke) must strictly adhere to a “temporal window” logic. This approach avoids the detrimental effects in the acute phase while harnessing the pathway’s prorepair potential in the subacute phase. Such a chronologically defined treatment strategy is mechanistically complementary to hyperacute thrombolytic therapy [47].

In models of developmental brain injury, this pathway exhibits a protective‐dominant pattern that diverges from its role in adult stroke. Neonatal hypoxic‐ischemic encephalopathy is usually caused by perinatal asphyxia, and about 50% of survivors may develop complications such as intellectual disability, cerebral palsy, or epilepsy [48]. In contrast to ischemic injury in the adult brain, C3a plays a protective role in models of neonatal hypoxic‐ischemic brain injury. Compared to wild‐type mice, mice expressing biologically active C3a under the control of the glial fibrillary acidic protein promoter (GFAP‐C3a) showed reduced brain tissue loss 3 weeks after injury. Intracerebroventricular injection of C3a 1 h after unilateral hypoxia‐ischemia can alleviate hypoxia‐ischemia–induced memory impairment, and this protective effect is dependent on C3aR [49]. Three years later, the team found that intranasal administration of C3a improved hypoxia‐ischemia–induced reactive gliosis in the hippocampus, which could also treat cognitive impairment caused by ischemia‐hypoxia [50] (Figure 2). This contrasts sharply with its detrimental effects in the acute phase of adult stroke, strongly suggesting that the developmental stage and maturity of the brain are critical determinants regulating the functional output of C3aR.

In contrast, within the context of chronic neurodegenerative diseases and pathologies such as epilepsy, this pathway consistently demonstrates a clear pathogenic role, persistently driving neuronal injury and disease progression.

In experimental status epilepticus, the activation of astrocytes requires microglia. C3 from astrocytes activates microglia through the C3aR, and the interaction between microglia and astrocytes promotes gliosis and neuronal injury following seizures [51]. In C3 and C3aR KO mice, microglia‐astrocyte interactions significantly decrease in response to status epilepticus, with markedly less histochemical evidence of neurodegeneration. Current research has demonstrated that in aging and Alzheimer’s disease (AD) mouse models, as well as in AD patients, levels of C3 and C3aR in the brain are elevated, and their inactivation can prevent age‐related functional decline and AD neuropathology [52–54]. In AD, the C3a/C3aR pathway mediates microtubule‐associated protein tau regulation and neuroinflammation via targeting STAT3. The expression of C3aR shows a negative correlation with cognitive function and a positive correlation with Braak staging [53]. Furthermore, genetic knockout or pharmacological inhibition of C3aR alleviates tau pathology, neuroinflammation, and Aβ‐associated synaptic loss. In another recent study, the same team noted that deficiency of C3aR alleviates hypoxia‐inducible factor‐1α–induced metabolic impairment, enhances the clustering of microglia around Aβ plaques, and augments their reactivity and phagocytic activity, thereby improving synaptic loss and cognitive function [10]. In small vessel disease and vascular dementia, upregulation of complement C3 expression activates C3aR on the surface of microglia, inducing aberrant microglial activation. This process mediates the redistribution of microglia in the striatum and the phagocytosis of myelin during chronic cerebral hypoperfusion, resulting in white matter injury. Notably, C3aRAs can ameliorate the associated cognitive decline [55]. Neuromyelitis optica (NMO) is a severe inflammatory autoimmune central nervous system disease. Dr. Wulongjun’s research group, in collaboration with Dr. Vanda Lennon, developed an innovative NMO mouse model. Using two‐photon in vivo imaging technology, they tracked the interactions between microglia and astrocytes in the spinal cord. Combined with NMO‐IgG, astrocytes promote microglia binding to astrocytes by releasing complement C3, while microglia release complement C1q and bind to adjacent neurons, causing neuronal injury [56]. In a neuron‐specific ARF1 knockout‐induced neurodegenerative mouse model, the C3‐C3aR1 pathway mediates the neurotoxicity of activated A1 astrocytes, which promote neuronal death. This has been validated in samples from neurodegenerative patients [57]. Collectively, this evidence indicates that inhibiting the C3a‐C3aR pathway holds clear therapeutic potential in chronic neuropathological states.

In summary, the “good versus evil” duality of the C3a‐C3aR pathway in the nervous system can be unified under a higher‐order “injury‐repair balance” framework. Its functional output is jointly determined by the acute/chronic nature of the insult, the developmental or aged state of the brain, and the potential of the local microenvironment to shift from inflammation toward repair. In adult stroke, the function of this pathway undergoes an obligatory temporal switch, with aging exacerbating its acute‐phase detrimental effects via mechanisms such as exosome‐mediated communication. In neonatal brain injury, the developing brain microenvironment may initiate repair programs earlier, allowing protective effects to dominate. In chronic degenerative diseases, persistent pathological stimuli lock the pathway into a destructive mode. This framework not only explains seemingly contradictory results across studies but also charts a course for precision therapy: Temporally precise intervention is required in conditions like stroke, with careful consideration of age‐related influences on treatment response, whereas sustained inhibition of the pathway is favored in neurodegenerative disorders. Future research should focus on elucidating the molecular switches that control these functional conversions and exploring the use of peripheral biomarkers (e.g., exosomes and GFAP) for the noninvasive assessment of central C3aR pathway status, paving the way for truly personalized neuroimmunotherapeutic strategies.

### 3.4. Autoimmune Disease

In autoimmune diseases such as psoriasis and neovascular macular fibrosis, the activation of the C3a‐C3aR pathway plays a protective or promotive role in tissue injury and disease progression. Wang Gang and his team first discovered that the expression of C3aR is significantly reduced in the epidermis of psoriatic lesions. C3aR exhibits a protective effect in imiquimod (IMQ) and IL‐23–induced psoriasis mouse models. Treatment with C3aR agonist drugs improved IMQ‐induced psoriasis‐like lesions in mice by inhibiting keratinocyte proliferation through the suppression of K6, K16, and K17 expression [58] (Figure 2). A study from the UK shows that mesothelial‐mesenchymal transition (MMT) is involved in macular fibrosis secondary to neovascularization, and TGF‐β and complement C3a (but not C5a) are potential inducers of MMT in macular fibrosis [59]. Systemic lupus erythematosus is also a common cause of secondary osteoporosis. In a study by Correa‐Rodríguez et al. involving 121 premenopausal and postmenopausal Caucasian women, it was found that 8.3% of the patients had osteoporosis, 52.1% had osteopenia, and bone density was negatively correlated with complement C3 levels [60].

### 3.5. Cancer: Tumor Microenvironment and Aberrant Repair

The C3a/C3aR pathway not only plays a crucial role in immune surveillance but also serves as a key accomplice in tumor immune evasion. The complement system not only plays an important role in immune surveillance but also participates in regulating tumor growth and immune evasion within the tumor microenvironment. The C3a‐C3aR pathway has been found in some solid tumors to facilitate immune evasion by promoting the formation of an immunosuppressive microenvironment, thus helping tumor cells escape the surveillance of the immune system. A domestic study showed that in tumor‐associated fibroblasts (CAFs), C3a‐C3aR signaling enhances the secretion of prometastatic cytokines and the expression of extracellular matrix components via the PI3K‐AKT pathway, promoting breast cancer metastasis [61]. In mouse models, genetic or pharmacological blockade of C3aR signaling effectively inhibited lung metastasis of breast cancer. Similar conclusions were drawn in glioma‐related studies. In human glioblastoma, both C3 and its receptor C3aR1 are associated with invasiveness and a shortened survival period. In mouse models, it was found that C3 is specifically present in hypoxic tumor regions, and C3a induces M2 polarization of cultured microglia and MPs in a C3aR‐dependent manner [62]. Another study revealed the mechanism by which the NFAT 1‐C3a‐C3aR pathway in tumor‐associated MPs (TAMs) activates M2‐like polarization positive feedback, promoting the malignant phenotype of glioma [6]. In osteosarcoma, complement activation related to the lectin pathway mediates C3aR‐dependent immunosuppression, promoting tumor progression, and a lack of C3aR in patients is associated with better clinical outcomes [63]. Overall, C3a can activate TAMs through C3aR, and these TAMs often exhibit immunosuppressive characteristics, promoting tumor growth and metastasis. Inhibiting C3aR activity can reactivate antitumor immune responses, a pathogenic mode characterized by a “tumor‐derived DAMP trigger + myeloid cell (TAM) hijacking” mechanism.

## 4. Therapeutic Strategies Targeting C3a‐C3aR: Challenges for Precision Intervention

Given the critical yet paradoxical roles of the C3a‐C3aR pathway across various diseases observed in preclinical and clinical sample studies, it represents a compelling preclinical therapeutic target. However, its inherent “double‐edged sword” nature dictates that intervention strategies cannot be simplified to mere inhibition or activation; moreover, most current findings are based on preclinical studies (animal experiments and in vitro studies), so intervention strategies, if applied to clinical practice, must constitute a highly context‐dependent precision medicine practice, which requires sufficient clinical evidence support.

The choice of therapeutic strategy depends entirely on the specific pathological background, forming the core logic of precision intervention.

Preclinical evidence supports the potential value of C3aRAs in acute‐phase ischemic stroke, AD, lupus nephritis, diabetic nephropathy, and as combination strategies with cancer immunotherapy. For instance, tool compounds like the small‐molecule antagonist SB 290157 have demonstrated efficacy across multiple disease models. Regarding C3aR agonists/C3a mimetics, the most robust preclinical evidence comes from the subacute phase following ischemic stroke (delayed administration), neonatal hypoxic–ischemic encephalopathy, and specific models of bacterial kidney infection. Agonists have also shown disease‐modifying phenotypes in psoriasis models. The dynamic evolution of disease means that the same patient may require opposing strategies at different stages. For example, in stroke patients, inhibition may be needed in the acute phase, while agonism could be beneficial in the subacute phase. Designing smart drugs or delivery regimens to achieve such temporal and cell‐specific control represents a major future challenge for medicinal chemistry and delivery systems.

A primary bottleneck in clinical translation is the lack of reliable predictive biomarkers (e.g., imaging connectivity and fluid C3a/GFAP levels) for patient stratification. Secondly, for central nervous system disorders, effective drug delivery (e.g., intranasal administration) still requires clinical validation. The foremost safety concern is that systemic inhibition may increase infection risk, while inappropriate agonism could promote fibrosis or tumor progression. In summary, despite encouraging preclinical data, advancing C3a‐C3aR–targeted therapies to the clinic must first address three major challenges: precise patient stratification, spatiotemporal‐specific modulation, and long‐term safety. Future translational research should focus on developing companion diagnostic biomarkers and conducting proof‐of‐concept clinical trials in well‐defined patient subgroups with clear pathological mechanisms.

## 5. Conclusion and Outlook

The C3a‐C3aR pathway plays a critical role in the onset and progression of various diseases, particularly in the fields of cardiovascular diseases, neurodegenerative diseases, autoimmune diseases, and tumors, where the mechanism of C3a‐C3aR is gradually being unveiled. Although current therapeutic strategies targeting C3aR are still in the early stages of development, it holds great potential as a multifunctional regulatory molecule in future disease treatments. Future research should further clarify the specific mechanisms of action of the C3a‐C3aR pathway in different disease contexts to provide stronger evidence for its clinical application.

## Funding

This study was supported by the Jiangsu Province Key Project for Cadre Healthcare (Grant BJ2407).

## Conflicts of Interest

The authors declare no conflicts of interest.

## Data Availability

Research data are not shared.

## References

[1] Merle N. S. , Church S. E. , Fremeaux-Bacchi V. , and Roumenina L. T. , Complement System Part I - Molecular Mechanisms of Activation and Regulation, Frontiers in Immunology. (2015) 6, 10.3389/fimmu.2015.00262, 2-s2.0-84935118905, 262. 26082779 PMC4451739

[2] Walport M. J. , Complement. First of Two Parts, New England Journal of Medicine. (2001) 344, no. 14, 1058–1066. 11287977 10.1056/NEJM200104053441406

[3] Zarantonello A. , Revel M. , Grunenwald A. , and Roumenina L. T. , C3 -Dependent Effector Functions of Complement, Immunological Reviews. (2023) 313, no. 1, 120–138, 10.1111/imr.13147. 36271889 PMC10092904

[4] Mathern D. R. and Heeger P. S. , Molecules Great and Small: The Complement System, Clinical Journal of the American Society of Nephrology. (2015) 10, no. 9, 1636–1650, 10.2215/CJN.06230614, 2-s2.0-84930053899. 25568220 PMC4559511

[5] Martin U. , Bock D. , and Arseniev L. , et al.The Human C3a Receptor Is Expressed on Neutrophils and Monocytes, But Not on B or T Lymphocytes, The Journal of Experimental Medicine. (1997) 186, no. 2, 199–207, 10.1084/jem.186.2.199, 2-s2.0-0030739649. 9221749 PMC2198980

[6] Zhang Y. , Song Y. , and Wang X. , et al.An NFAT1-C3a-C3aR Positive Feedback Loop in Tumor-Associated Macrophages Promotes a Glioma Stem Cell Malignant Phenotype, Cancer Immunology Research. (2024) 12, no. 3, 363–376, 10.1158/2326-6066.CIR-23-0418. 38289255

[7] Monteran L. , Ershaid N. , and Doron H. , et al.Chemotherapy-Induced Complement Signaling Modulates Immunosuppression and Metastatic Relapse in Breast Cancer, Nature Communications. (2022) 13, no. 1, 10.1038/s41467-022-33598-x, 5797. PMC9527249 36184683

[8] Propson N. E. , Roy E. R. , Litvinchuk A. , Köhl J. , and Zheng H. , Endothelial C3a Receptor Mediates Vascular Inflammation and Blood-Brain Barrier Permeability During Aging, The Journal of Clinical Investigation. (2021) 131, no. 1, 10.1172/JCI140966. PMC7773352 32990682

[9] Zhang Q. , Bin S. , and Budge K. , et al.C3aR-Initiated Signaling Is a Critical Mechanism of Podocyte Injury in Membranous Nephropathy, JCI Insight. (2024) 9, no. 4, 10.1172/jci.insight.172976. PMC11143932 38227377

[10] Gedam M. , Comerota M. M. , and Propson N. E. , et al.Complement C3aR Depletion Reverses HIF-1α-Induced Metabolic Impairment and Enhances Microglial Response to Aβ Pathology, The Journal of Clinical Investigation. (2023) 133, no. 12, 10.1172/JCI167501. PMC10266793 37317973

[11] Yadav M. K. , Maharana J. , and Yadav R. , et al.Molecular Basis of Anaphylatoxin Binding, Activation, and Signaling Bias at Complement Receptors, Cell. (2023) 186, no. 22, 4956–4973.e21, 10.1016/j.cell.2023.09.020. 37852260 PMC7615941

[12] Liszewski M. K. , Kolev M. , and Le Friec G. , et al.Intracellular Complement Activation Sustains T Cell Homeostasis and Mediates Effector Differentiation, Immunity. (2013) 39, no. 6, 1143–1157, 10.1016/j.immuni.2013.10.018, 2-s2.0-84890226478. 24315997 PMC3865363

[13] Freeley S. , Kemper C. , and Le Friec G. , The “Ins and Outs” of Complement-Driven Immune Responses, Immunological Reviews. (2016) 274, no. 1, 16–32, 10.1111/imr.12472, 2-s2.0-84992450133. 27782335 PMC5102160

[14] Niyonzima N. , Rahman J. , and Kunz N. , et al.Mitochondrial C5aR1 Activity in Macrophages Controls IL-1β Production Underlying Sterile Inflammation, Science Immunology. (2021) 6, no. 66, 10.1126/sciimmunol.abf2489, eabf2489. 34932384 PMC8902698

[15] West E. E. and Kemper C. , Complosome - The Intracellular Complement System, Nature Reviews Nephrology. (2023) 19, no. 7, 426–439, 10.1038/s41581-023-00704-1. 37055581 PMC10100629

[16] Kremlitzka M. , Nowacka A. A. , Mohlin F. C. , Bompada P. , De Marinis Y. , and Blom A. M. , Interaction of Serum-Derived and Internalized C3 With DNA in Human B Cells—A Potential Involvement in Regulation of Gene Transcription, Frontiers in Immunology. (2019) 10, 10.3389/fimmu.2019.00493, 2-s2.0-85064230388, 493. 30941132 PMC6433827

[17] King B. C. , Kulak K. , and Krus U. , et al.Complement Component C3 Is Highly Expressed in Human Pancreatic Islets and Prevents β Cell Death via ATG16L1 Interaction and Autophagy Regulation, Cell Metabolism. (2019) 29, no. 1, 202–210.e6, 10.1016/j.cmet.2018.09.009, 2-s2.0-85059234016. 30293775

[18] Liu L. , Zhang Y. , and Duan X. , et al.C3a, C5a Renal Expression and Their Receptors are Correlated to Severity of IgA Nephropathy, Journal of Clinical Immunology. (2014) 34, no. 2, 224–232, 10.1007/s10875-013-9970-6, 2-s2.0-84898896776. 24327134

[19] Wang Z. , Xie X. , and Li J. , et al.Complement Activation Is Associated With Crescents in IgA Nephropathy, Frontiers in Immunology. (2021) 12, 10.3389/fimmu.2021.676919, 676919. 34594322 PMC8477028

[20] Zhang Y. , Yan X. , and Zhao T. , et al.Targeting C3a/C5a Receptors Inhibits Human Mesangial Cell Proliferation and Alleviates Immunoglobulin A Nephropathy in Mice, Clinical and Experimental Immunology. (2017) 189, no. 1, 60–70, 10.1111/cei.12961, 2-s2.0-85017412802. 28295247 PMC5461107

[21] Bao L. , Osawe I. , Haas M. , and Quigg R. J. , Signaling Through Up-Regulated C3a Receptor Is Key to the Development of Experimental Lupus Nephritis, The Journal of Immunology. (2005) 175, no. 3, 1947–1955, 10.4049/jimmunol.175.3.1947, 2-s2.0-22544469404. 16034139

[22] Weng M. , Wu X. , and Rao S. , et al.C3a/C3aR Axis Is Involved in Diabetic Kidney Injury by Regulating Podocyte Mitophagy in Diabetic Nephropathy, International Journal of Molecular Medicine. (2025) 56, no. 6, 1–15, 10.3892/ijmm.2025.5664. PMC12549072 41104892

[23] Yang Y. , Wang C. , and Jin L. , et al.IgG4 Anti-Phospholipase A2 Receptor Might Activate Lectin and Alternative Complement Pathway Meanwhile in Idiopathic Membranous Nephropathy: An Inspiration From a Cross-Sectional Study, Immunologic Research. (2016) 64, no. 4, 919–930, 10.1007/s12026-016-8790-1, 2-s2.0-84983087361. 26837241

[24] Han R. , Hu S. , and Qin W. , et al.C3a and suPAR Drive Versican V1 Expression in Tubular Cells of Focal Segmental Glomerulosclerosis, JCI Insight. (2019) 4, no. 13, 10.1172/jci.insight.130986, 2-s2.0-85070658760. PMC6629242 31292294

[25] Li X.-Q. , Chang D.-Y. , Chen M. , and Zhao M.-H. , Complement Activation in Patients With Diabetic Nephropathy, Diabetes & Metabolism. (2019) 45, no. 3, 248–253, 10.1016/j.diabet.2018.04.001, 2-s2.0-85046782927. 29729954

[26] Simone S. , Rascio F. , and Castellano G. , et al.Complement-Dependent NADPH Oxidase Enzyme Activation in Renal Ischemia/Reperfusion Injury, Free Radical Biology and Medicine. (2014) 74, 263–273, 10.1016/j.freeradbiomed.2014.07.003, 2-s2.0-84905380684. 25017967

[27] Curci C. , Castellano G. , and Stasi A. , et al.Endothelial-to-Mesenchymal Transition and Renal Fibrosis in Ischaemia/Reperfusion Injury Are Mediated by Complement Anaphylatoxins and Akt Pathway, Nephrology, Dialysis, Transplantation: Official Publication of the European Dialysis and Transplant Association - European Renal Association. (2014) 29, no. 4, 799–808. 24463188 10.1093/ndt/gft516

[28] Bao L. , Wang Y. , Haas M. , and Quigg R. J. , Distinct Roles for C3a and C5a in Complement-Induced Tubulointerstitial Injury, Kidney International. (2011) 80, no. 5, 524–534, 10.1038/ki.2011.158, 2-s2.0-84863010724. 21677637

[29] Tang Z. , Lu B. , Hatch E. , Sacks S. H. , and Sheerin N. S. , C3a Mediates Epithelial-to-Mesenchymal Transition in Proteinuric Nephropathy, Journal of the American Society of Nephrology. (2009) 20, no. 3, 593–603, 10.1681/ASN.2008040434, 2-s2.0-62149135267. 19158354 PMC2653680

[30] Mueller-Ortiz S. L. , Morales J. E. , and Wetsel R. A. , The Receptor for the Complement C3a Anaphylatoxin (C3aR) Provides Host Protection Against *Listeria monocytogenes*-Induced Apoptosis, Journal of Immunology. (2014) 193, no. 3, 1278–1289. 10.4049/jimmunol.1302787 PMC4122265 24981453

[31] Mueller-Ortiz S. L. , Calame D. G. , and Shenoi N. , et al.The Complement Anaphylatoxins C5a and C3a Suppress IFN-β Production in Response to *Listeria monocytogenes* by Inhibition of the Cyclic Dinucleotide-Activated Cytosolic Surveillance Pathway, Journal of Immunology. (2017) 198, no. 8, 3237–3244. 10.4049/jimmunol.1601420 PMC5398560 28275134

[32] Wu K.-Y. , Zhang T. , and Zhao G.-X. , et al.The C3a/C3aR Axis Mediates Anti-Inflammatory Activity and Protects Against Uropathogenic E Coli-Induced Kidney Injury in Mice, Kidney International. (2019) 96, no. 3, 612–627, 10.1016/j.kint.2019.03.005, 2-s2.0-85065924407. 31133456

[33] Engström G. , Hedblad B. , and Janzon L. , et al.Complement C3 and C4 in Plasma and Incidence of Myocardial Infarction and Stroke: A Population-Based Cohort Study., European Journal of Cardiovascular Prevention and Rehabilitation: Official Journal of the European Society of Cardiology Working Groups on Epidemiology & Prevention and Cardiac Rehabilitation and Exercise Physiology. (2007) 14, no. 3, 392–397. 10.1097/01.hjr.0000244582.30421.b2 17568238

[34] Palikhe A. , Sinisalo J. , and Seppänen M. , et al.Serum Complement C3/C4 Ratio, a Novel Marker for Recurrent Cardiovascular Events, The American Journal of Cardiology. (2007) 99, no. 7, 890–895, 10.1016/j.amjcard.2006.11.034, 2-s2.0-33947608775. 17398178

[35] Nijmeijer R. , Krijnen P. A. J. , and Assink J. , et al.C-Reactive Protein and Complement Depositions in Human Infarcted Myocardium Are More Extensive in Patients With Reinfarction or Upon Treatment With Reperfusion, European Journal of Clinical Investigation. (2004) 34, no. 12, 803–810, 10.1111/j.1365-2362.2004.01425.x, 2-s2.0-10644222813. 15606722

[36] Hill J. H. and Ward P. A. , The Phlogistic Role of C3 Leukotactic Fragments in Myocardial Infarcts of Rats, The Journal of Experimental Medicine. (1971) 133, no. 4, 885–900, 10.1084/jem.133.4.885, 2-s2.0-0015042034. 4993831 PMC2138969

[37] Hartmann K. , Henz B. M. , and Krüger-Krasagakes S. , et al.C3a and C5a Stimulate Chemotaxis of Human Mast Cells, Blood. (1997) 89, no. 8, 2863–2870, 10.1182/blood.V89.8.2863. 9108406

[38] Zhang C. , Wang C. , and Li Y. , et al.Complement C3a Signaling Facilitates Skeletal Muscle Regeneration by Regulating Monocyte Function and Trafficking, Nature Communications. (2017) 8, no. 1, 10.1038/s41467-017-01526-z, 2-s2.0-85037738026, 2078. PMC5727192 29233958

[39] Daffern P. J. , Pfeifer P. H. , Ember J. A. , and Hugli T. E. , C3a Is a Chemotaxin for Human Eosinophils but Not for Neutrophils. I. C3a Stimulation of Neutrophils Is Secondary to Eosinophil Activation, The Journal of Experimental Medicine. (1995) 181, no. 6, 2119–2127, 10.1084/jem.181.6.2119, 2-s2.0-0029000948. 7760001 PMC2192052

[40] Sauter R. J. , Sauter M. , and Reis E. S. , et al.Functional Relevance of the Anaphylatoxin Receptor C3aR for Platelet Function and Arterial Thrombus Formation Marks an Intersection Point Between Innate Immunity and Thrombosis, Circulation. (2018) 138, no. 16, 1720–1735, 10.1161/CIRCULATIONAHA.118.034600, 2-s2.0-85053515062. 29802205 PMC6202244

[41] Trendelenburg M. , Stallone F. , and Pershyna K. , et al.Complement Activation Products in Acute Heart Failure: Potential Role in Pathophysiology, Responses to Treatment and Impacts on Long-Term Survival, European Heart Journal: Acute Cardiovascular Care. (2018) 7, no. 4, 348–357, 10.1177/2048872617694674, 2-s2.0-85052808562. 29064269

[42] Gombos T. , Förhécz Z. , and Pozsonyi Z. , et al.Complement Anaphylatoxin C3a as a Novel Independent Prognostic Marker in Heart Failure, Clinical Research in Cardiology. (2012) 101, no. 8, 607–615, 10.1007/s00392-012-0432-6, 2-s2.0-84865785162. 22373875

[43] Ito S. , Hashimoto H. , and Yamakawa H. , et al.The Complement C3-Complement Factor D-C3a Receptor Signalling Axis Regulates Cardiac Remodelling in Right Ventricular Failure, Nature Communications. (2022) 13, no. 1, 10.1038/s41467-022-33152-9, 5409. PMC9478115 36109509

[44] Li R. G. , Li X. , and Morikawa Y. , et al.YAP Induces a Neonatal-Like Pro-Renewal Niche in the Adult Heart, Nature Cardiovascular Research. (2024) 3, no. 3, 283–300, 10.1038/s44161-024-00428-w. PMC10954255 38510108

[45] Komeno M. , Pang X. , and Shimizu A. , et al.Cardio- and Reno-Protective Effects of Dipeptidyl Peptidase III in Diabetic Mice, Journal of Biological Chemistry. (2021) 296, 10.1016/j.jbc.2021.100761, 100761. 33971198 PMC8167299

[46] Zhang H. , Lin S. , and McElroy C. L. , et al.Circulating Pro-Inflammatory Exosomes Worsen Stroke Outcomes in Aging, Circulation Research. (2021) 129, no. 7, e121–e40, 10.1161/CIRCRESAHA.121.318897. 34399581 PMC8448978

[47] Stokowska A. , Aswendt M. , and Zucha D. , et al.Complement C3a Treatment Accelerates Recovery After Stroke via Modulation of Astrocyte Reactivity and Cortical Connectivity, Journal of Clinical Investigation. (2023) 133, no. 10, 10.1172/JCI162253. PMC10178843 36995772

[48] Pekna M. , Stokowska A. , and Pekny M. , Targeting Complement C3a Receptor to Improve Outcome After Ischemic Brain Injury, Neurochemical Research. (2021) 46, no. 10, 2626–2637, 10.1007/s11064-021-03419-6. 34379293 PMC8437837

[49] Järlestedt K. , Rousset C. I. , and Ståhlberg A. , et al.Receptor for Complement Peptide C3a: A Therapeutic Target for Neonatal Hypoxic-Ischemic Brain Injury, The FASEB Journal. (2013) 27, no. 9, 3797–3804, 10.1096/fj.13-230011, 2-s2.0-84883364402. 23737250

[50] Morán J. , Stokowska A. , Walker F. R. , Mallard C. , Hagberg H. , and Pekna M. , Intranasal C3a Treatment Ameliorates Cognitive Impairment in a Mouse Model of Neonatal Hypoxic-Ischemic Brain Injury, Experimental Neurology. (2017) 290, 74–84, 10.1016/j.expneurol.2017.01.001, 2-s2.0-85009412324. 28062175

[51] Wei Y. , Chen T. , and Bosco D. B. , et al.The Complement C3-C3aR Pathway Mediates Microglia-Astrocyte Interaction Following Status Epilepticus, Glia. (2021) 69, no. 5, 1155–1169, 10.1002/glia.23955. 33314324 PMC7936954

[52] Lian H. , Yang L. , and Cole A. , et al.NFκB-Activated Astroglial Release of Complement C3 Compromises Neuronal Morphology and Function Associated With Alzheimer’s Disease, Neuron. (2015) 85, no. 1, 101–115, 10.1016/j.neuron.2014.11.018, 2-s2.0-84920841382. 25533482 PMC4289109

[53] Litvinchuk A. , Wan Y.-W. , and Swartzlander D. B. , et al.Complement C3aR Inactivation Attenuates Tau Pathology and Reverses an Immune Network Deregulated in Tauopathy Models and Alzheimer’s Disease, Neuron. (2018) 100, no. 6, 1337–1353.e5, 10.1016/j.neuron.2018.10.031, 2-s2.0-85058511942. 30415998 PMC6309202

[54] Wu T. , Dejanovic B. , and Gandham V. D. , et al.Complement C3 Is Activated in Human AD Brain and Is Required for Neurodegeneration in Mouse Models of Amyloidosis and Tauopathy, Cell Reports. (2019) 28, no. 8, 2111–2123.e6, 10.1016/j.celrep.2019.07.060, 2-s2.0-85070220949. 31433986

[55] Zhang L.-Y. , Pan J. , and Mamtilahun M. , et al.Microglia Exacerbate White Matter Injury via Complement C3/C3aR Pathway After Hypoperfusion, Theranostics. (2020) 10, no. 1, 74–90, 10.7150/thno.35841. 31903107 PMC6929610

[56] Chen T. , Lennon V. A. , and Liu Y. U. , et al.Astrocyte-Microglia Interaction Drives Evolving Neuromyelitis Optica Lesion, Journal of Clinical Investigation. (2020) 130, no. 8, 4025–4038, 10.1172/JCI134816. 32568214 PMC7410082

[57] Wang G. , Jin S. , and Liu J. , et al.A Neuron-Immune Circuit Regulates Neurodegeneration in the Hindbrain and Spinal Cord of Arf1-Ablated Mice, National Science Review. (2023) 10, no. 12, 10.1093/nsr/nwad222, nwad222. 38239560 PMC10794899

[58] Qiao P. , Zhi D. , and Yu C. , et al.Activation of the C3A Anaphylatoxin Receptor Inhibits Keratinocyte Proliferation by Regulating Keratin 6, Keratin 16, and Keratin 17 in Psoriasis, The FASEB Journal. (2022) 36, no. 5, 10.1096/fj.202101458R, e22322. 35429062

[59] Little K. , Llorián-Salvador M. , and Tang M. , et al.Macrophage to Myofibroblast Transition Contributes to Subretinal Fibrosis Secondary to Neovascular Age-Related Macular Degeneration, Journal of Neuroinflammation. (2020) 17, no. 1, 10.1186/s12974-020-02033-7, 355. 33239022 PMC7690191

[60] Correa-Rodríguez M. , Pocovi-Gerardino G. , and Callejas-Rubio J. L. , et al.Disease Damage Accrual and Low Bone Mineral Density in Female Patients With Systemic Lupus Erythematosus, Biological Research for Nursing. (2021) 23, no. 4, 575–583, 10.1177/10998004211005550. 33787331

[61] Shu C. , Zha H. , and Long H. , et al.C3a-C3aR Signaling Promotes Breast Cancer Lung Metastasis via Modulating Carcinoma Associated Fibroblasts, Journal of Experimental & Clinical Cancer Research. (2020) 39, no. 1, 10.1186/s13046-019-1515-2, 11. 31931851 PMC6958674

[62] Rosberg R. , Smolag K. I. , and Sjölund J. , et al.Hypoxia-Induced Complement Component 3 Promotes Aggressive Tumor Growth in the Glioblastoma Microenvironment, JCI Insight. (2024) 9, no. 19, 10.1172/jci.insight.179854. PMC11466187 39172519

[63] Magrini E. , Di Marco S. , and Mapelli S. N. , et al.Complement Activation Promoted by the Lectin Pathway Mediates C3aR-Dependent Sarcoma Progression and Immunosuppression, Nature Cancer. (2021) 2, no. 2, 218–232, 10.1038/s43018-021-00173-0. 34505065 PMC8425276

